# Vascular risks in external sinus lifts: an anatomical approach

**DOI:** 10.1080/07853890.2021.1897405

**Published:** 2021-09-28

**Authors:** P. Rodrigues, B. Saldanha, P. Sottomayor, R. Vaz

**Affiliations:** a Implantology Consultation at I.U.E.M.’s Dental Clinic, Egas Moniz CRL, Caparica, Portugal; b Voluntary Formation in the Implantology Consultation at I.U.E.M.’s Dental Clinic, Egas Moniz CRL, Caparica, Portugal

## Abstract

**Introduction:**

In the anterolateral wall of the maxillary sinus, an osseous canal can be observed, corresponding to the anastomosis between arterial branches of the posterior superior alveolar artery and the infraorbitary artery [1]. From this anastomosis, mostly known as Antral artery, its several branches allow for the vascularisation of the sinus mucous membrane, periosteum and the anterolateral bone wall of the sinus [2]. Surgical approach of this region, including antrostomy for external sinus lift of the maxillary sinus, can result in intraoperative bleeding due to vascular lesion during the osteotomy [3]. Current radiological 3D modelling of the sinus anatomy, as well as solid knowledge of sinus anatomy are of the upmost importance to prevent vascular complications during these surgical procedures in this area. The objective of this presentation is to graphically review and demonstrate anatomical concepts on sinus anatomy, through live and interactive *a viva voce* anatomical black board and chalk schematic drawings, based on the general narrative description of sinus anatomy, as well as anatomical findings regarding antral artery position in cadaveric studies [4–7].

**Materials and Methods:**

Medline (Pubmed) search using the following: “vascularization maxillary sinus cadavers”, completed with the revision of the bibliographical references of the selected articles in order to identify further relevant studies. Inclusion criteria: (1) Published between 2008 and 2015. (2) Cadaver Studies. (3) Studies that evaluate the position of the anatomical position of the anastomosis between arterial branches of the posterior superior alveolar artery and the infraorbitary artery. Exclusion Criteria: (1) Non cadaveric studies. (2) Foetal and paediatric studies.

**Results:**

Eight articles were selected.

**Discussion and conclusions:**

The vascular anastomosis is frequently found in the lateral wall of the maxillary sinus. Its position and depth are variable, as well as its trajectory and morphology. Pre-surgical determination of its precise anatomical variations is of the utmost importance to avoid vascular damage and complications during external sinus lifts osteotomies.
